# Anatomy of Inferior Mesenteric Artery in Fetuses

**DOI:** 10.1155/2016/5846578

**Published:** 2016-05-22

**Authors:** Ayesha Nuzhat

**Affiliations:** Faculty of Medicine, King Fahad Medical City, P.O. Box 59046, Riyadh 11525, Saudi Arabia

## Abstract

*Aim*. To analyze Inferior Mesenteric Artery in fetuses through its site of origin, length, diameter, and variation of its branches.* Method*. 100 fetuses were collected from various hospitals in Warangal at Kakatiya Medical College in Andhra Pradesh, India, and were divided into two groups, group I (second-trimester fetuses) and group II (third-trimester fetuses), followed by dissection.* Result*.* (1) Site of Origin*. In group I fetuses, origin of Inferior Mesenteric Artery was at third lumbar vertebra in 33 out of 34 fetuses (97.2%). In one fetus it was at first lumbar vertebra, 2.8%. In all group II fetuses, origin of Inferior Mesenteric Artery was at third lumbar vertebra.* (2) Length*. In group I fetuses it ranged between 18 and 30 mm, average being 24 mm except in one fetus where it was 48 mm. In group II fetuses the length ranged from 30 to 34 mm, average being 32 mm.* (3) Diameter*. In group I fetuses it ranged from 0.5 to 1 mm, and in group II fetuses it ranged from 1 to 2 mm, average being 1.5 mm.* (4) Branches*. Out of 34 fetuses of group I, 4 fetuses showed variation. In one fetus left colic artery was arising from abdominal aorta, 2.9%. In 3 fetuses, Inferior Mesenteric Artery was giving a branch to left kidney, 8.8%. Out of 66 fetuses in group II, 64 had normal branching. In one fetus left renal artery was arising from Inferior Mesenteric Artery, 1.5%, and in another fetus one accessory renal artery was arising from Inferior Mesenteric Artery and entering the lower pole of left kidney.* Conclusion*. Formation, course, and branching pattern of an artery depend on development and origin of organs to attain the actual adult position.

## 1. Introduction

**Figure 1:**
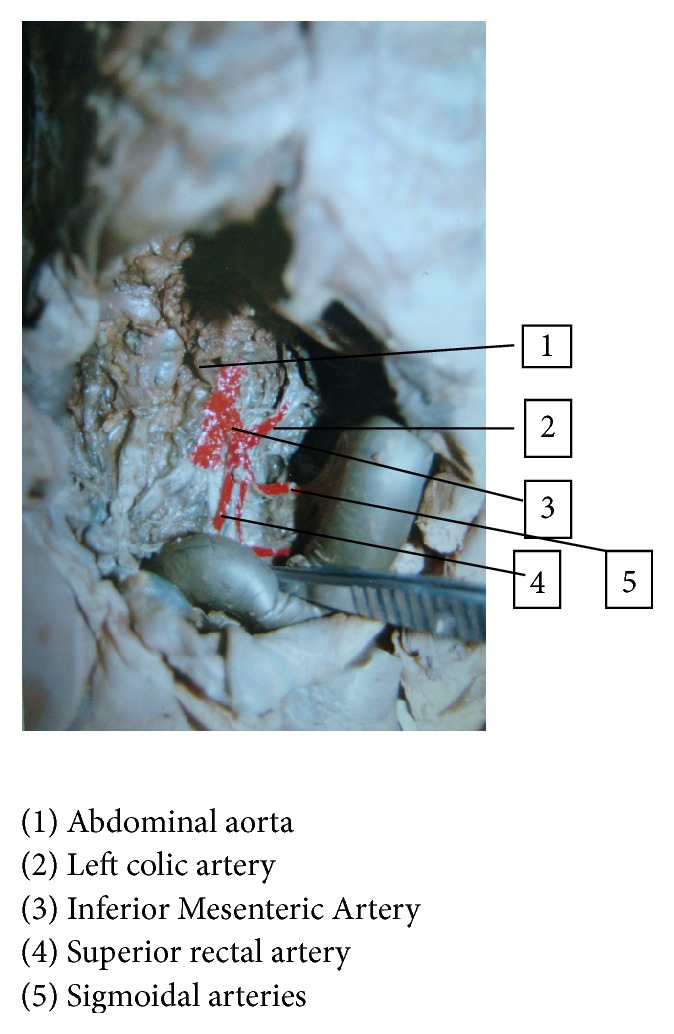
Inferior Mesenteric Artery and its branches, normal in fetus.

**Figure 2:**
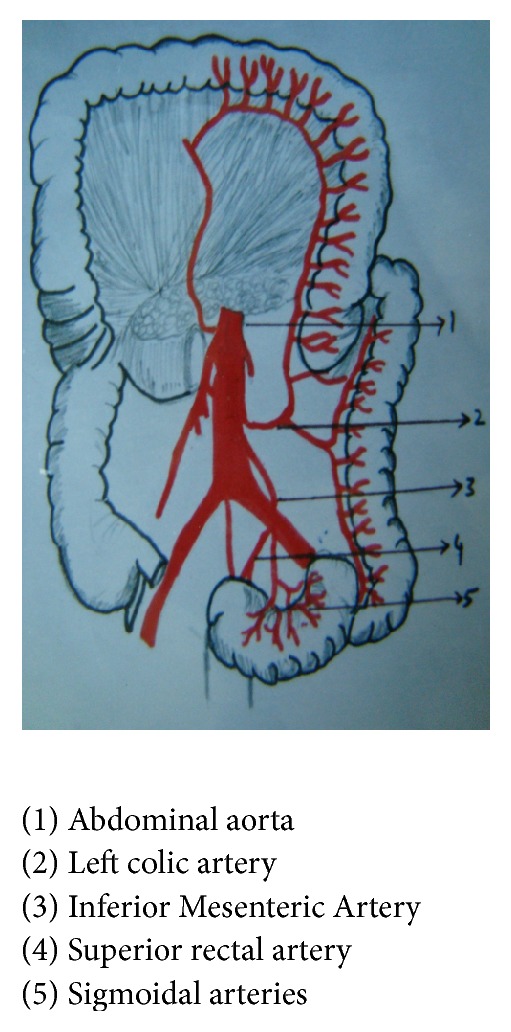
Inferior Mesenteric Artery and its branches, normal, sketch.

The Inferior Mesenteric Artery (Figures 1 and 2) is the unpaired visceral branch of abdominal aorta supplying the derivatives of hindgut, namely, leaving one-third of transverse colon, descending and pelvic colon, rectum, and upper part of anal canal [1]. Knowledge of the morphological variations of the artery is important not only from the anatomical, but also from the surgical point of view and variations in fetuses have been infrequently mentioned in the anatomic literature [2]. This study reveals the variations of Inferior Mesenteric Artery and its branches in fetuses of different age groups. Detailed knowledge of Inferior Mesenteric Artery and its branches and their frequency of variation will enable pediatric and vascular surgeons to plan for surgery in the newborn and to prevent surgical errors and complications. Radiologists can also revive information regarding the variations in vasculature of Inferior Mesenteric Artery and pass on its implications to other physicians, especially the oncologists who deal with colon and rectal cancer.

## 2. Materials and Method

One hundred fetuses between age groups of four to nine months were collected at Kakatiya Medical College, from various hospitals in Warangal, Andhra Pradesh, India. Approval for the study was taken by the institutional review board of the university in Andhra Pradesh, India.

All fetuses were either stillborn or aborted. Group I were second-trimester fetuses and group II were third-trimester fetuses. Biparietal diameter, age, and weight of all the fetuses were noted. The fetuses were dissected to expose Inferior Mesenteric Artery and then observed for its site of origin, length, diameter, and variation of its branches. 50 cc of 10% formalin was injected into the abdomen. All fetuses were subsequently preserved in 10% formalin. A median incision extending from xiphisternal joint to superior margin of pubic symphysis and a transverse incision extending from xiphisternal joint until the midaxillary line were made. Lower down, a transverse incision extending from superior margin of pubic symphysis along the line of inguinal ligament to the highest curvature of iliac crest was made. The incision was then extended into all the layers of the anterior abdominal wall, and flaps were reflected laterally to expose the abdominal organs. The abdomen was eviscerated (liver, stomach, and spleen were removed). The small intestine with its mesentery was turned to the right to remove the left layer of mesentery and a layer of sigmoid mesocolon to expose the Inferior Mesenteric Artery.

Inferior Mesenteric Artery was observed for its site of origin, length, diameter, and variation of its branches. The normal course and variations were noted and photographed, if necessary, after drying and painting the arteries of the specimens.

## 3. Observation and Results

The Inferior Mesenteric Artery studied under the following parameters revealed the following.

### 3.1. Site of Origin

Site of origin of Inferior Mesenteric Artery in relation to lumbar vertebra was noted.

**Figure 3:**
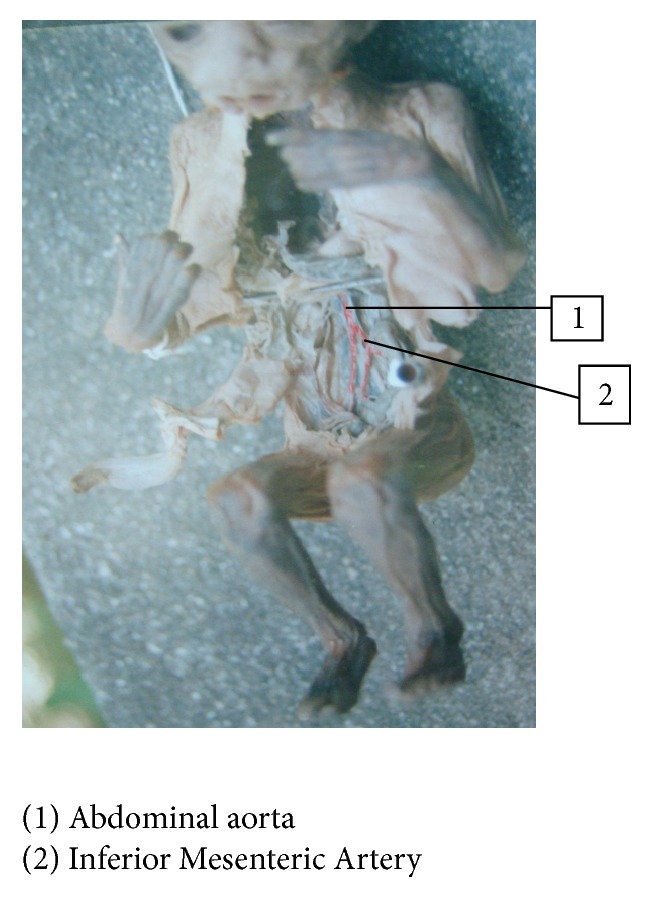
High origin of Inferior Mesenteric Artery from abdominal aorta at L1 in fetus.

**Figure 4:**
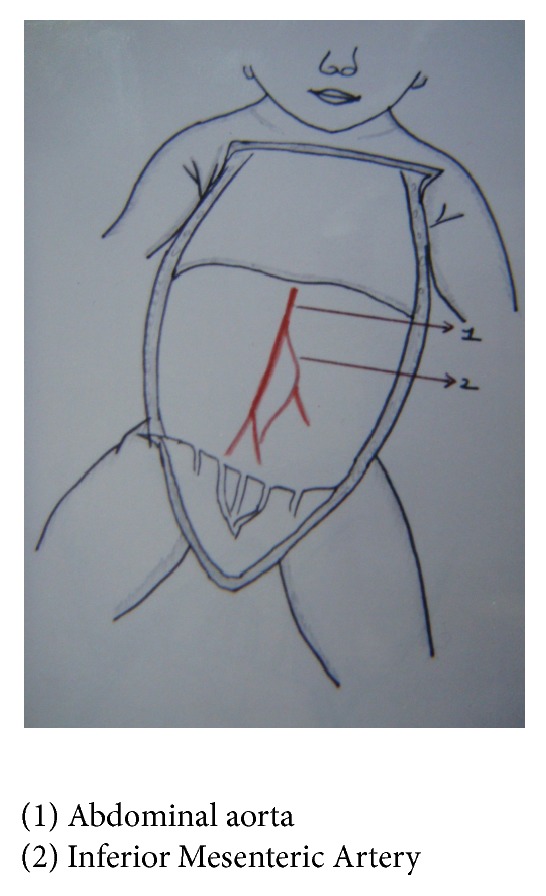
High origin of Inferior Mesenteric Artery from abdominal aorta at L1, sketch.

In group I fetuses, origin of Inferior Mesenteric Artery was at third lumbar vertebra in 33 out of 34 fetuses—97.2%. In one fetus, it was at first lumbar vertebra, fetus number 22: BPD—6.5 cms, age—24 weeks, and weight—600 gms, 2.8% (Figures 3 and 4).

In all group II fetuses, origin of Inferior Mesenteric Artery was at third lumbar vertebra.

### 3.2. Length

Inferior Mesenteric Artery was measured from its point of origin on aorta to point of division into superior rectal and last sigmoidal artery.

In group I fetuses it ranged between 18 and 30 mm, average being 24 mm except in one fetus where it was 48 mm, fetus number 22: BPD—6.5 cms, age—24 weeks, and weight—600 gms (Figures 3 and 4). In group II fetuses, the length ranged from 30 to 34 mm, average being 32 mm.

### 3.3. Diameter

The diameter of Inferior Mesenteric Artery in group I fetuses ranged from 0.5 to 1 mm and in group II fetuses ranged from 1 to 2 mm, average being 1.5 mm.

### 3.4. Branches

The branches observed were left colic, sigmoidal, and superior rectal artery.

Out of 34 fetuses of group I, 4 fetuses showed variation.

**Figure 5:**
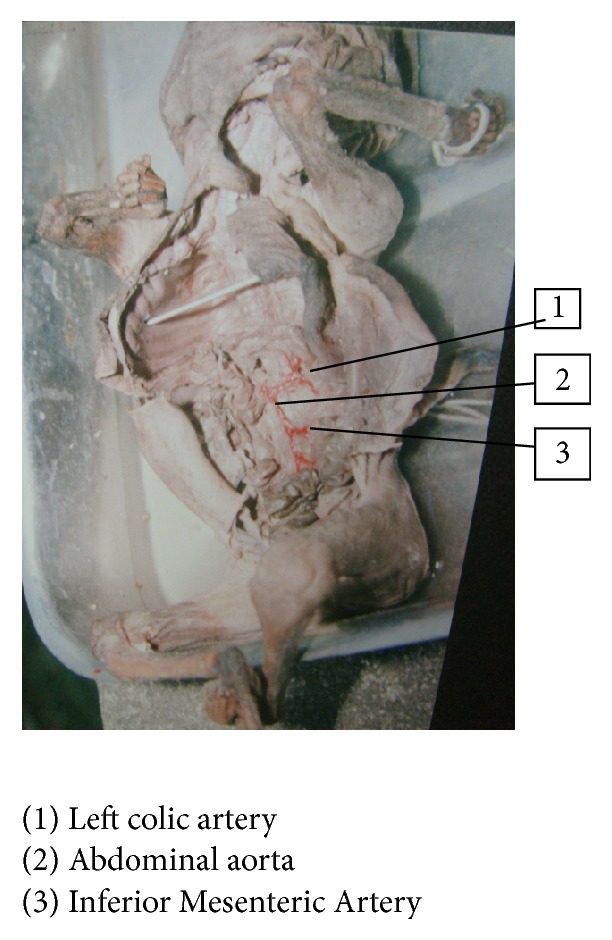
Origin of left colic artery from abdominal aorta.

**Figure 6:**
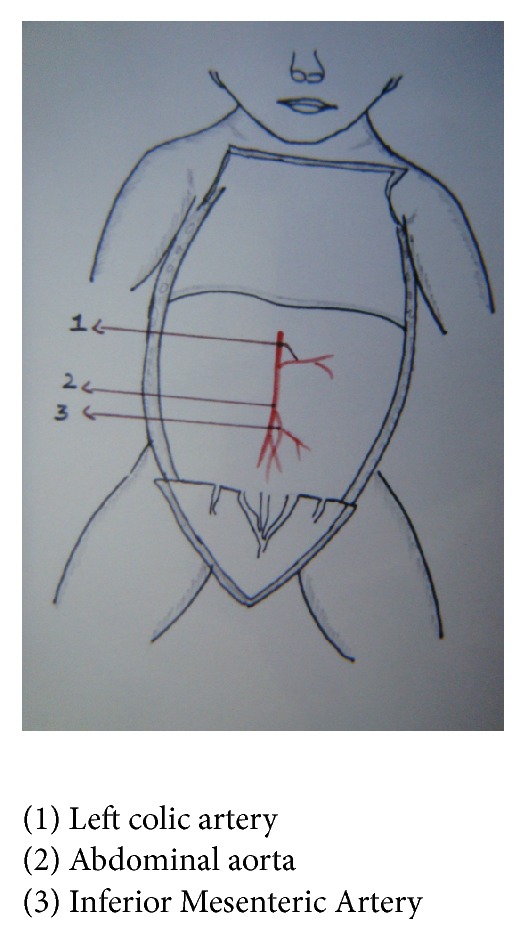
Origin of left colic artery from abdominal aorta.

In one fetus, left colic artery was arising from abdominal aorta—2.9%, fetus number 14: BPD—6 cms, age—23 weeks, and weight—1600 gms (Figures 5 and 6). In 3 fetuses, Inferior Mesenteric Artery was giving a branch to left kidney, 8.8%, fetus number 17: BPD—4.5 cms, age—17 weeks, and weight—175 gms; fetus number 90: BPD—4 cms, age—20 weeks, and weight—500 gms; and fetus number 54: BPD—7 cms, age—20 weeks, and weight—950 gms, respectively.

Out of 66 fetuses in group II, 64 had normal branching.

**Figure 7:**
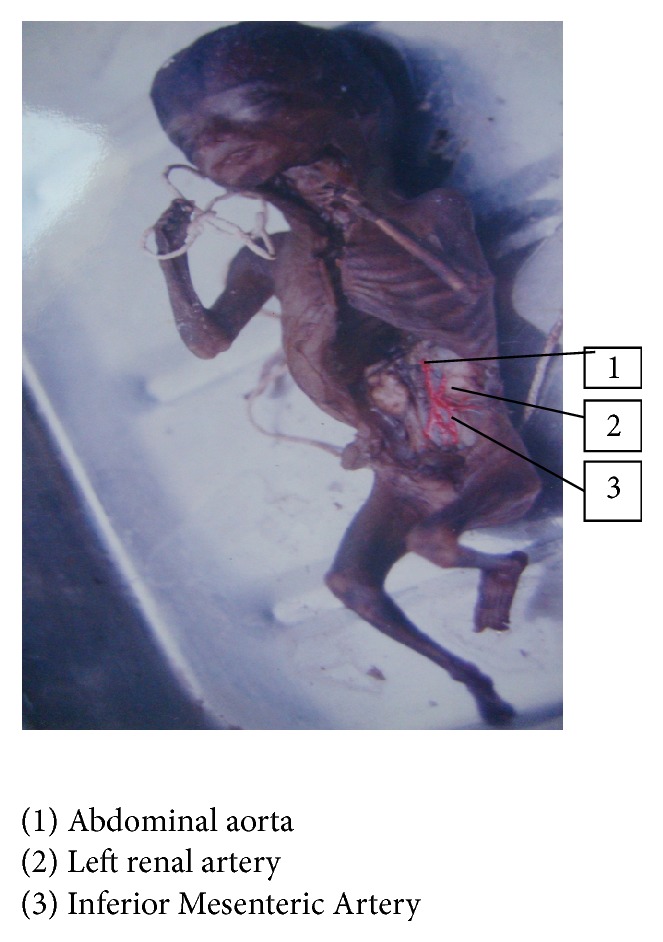
Origin of left renal artery from Inferior Mesenteric Artery, fetus.

**Figure 8:**
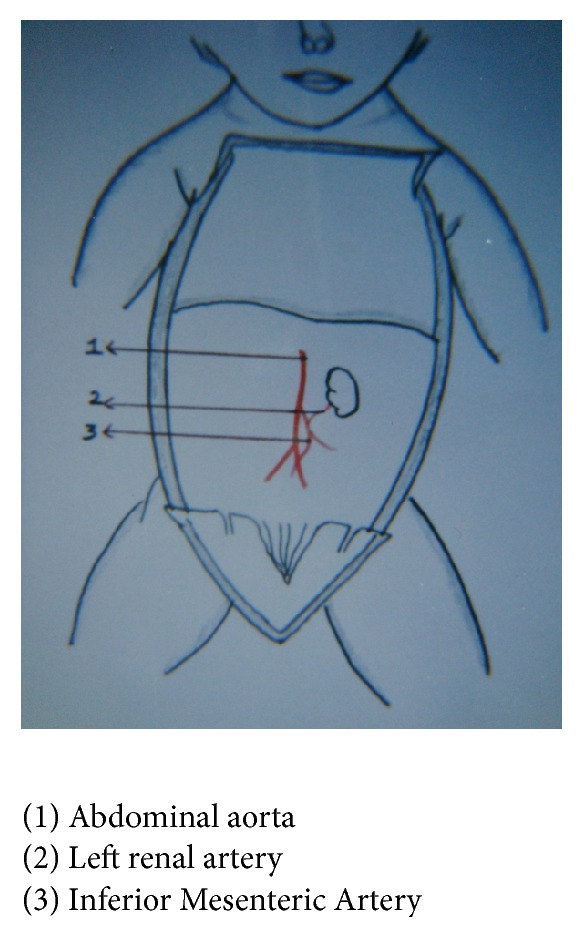
Origin of left renal artery from Inferior Mesenteric Artery, sketch.

**Figure 9:**
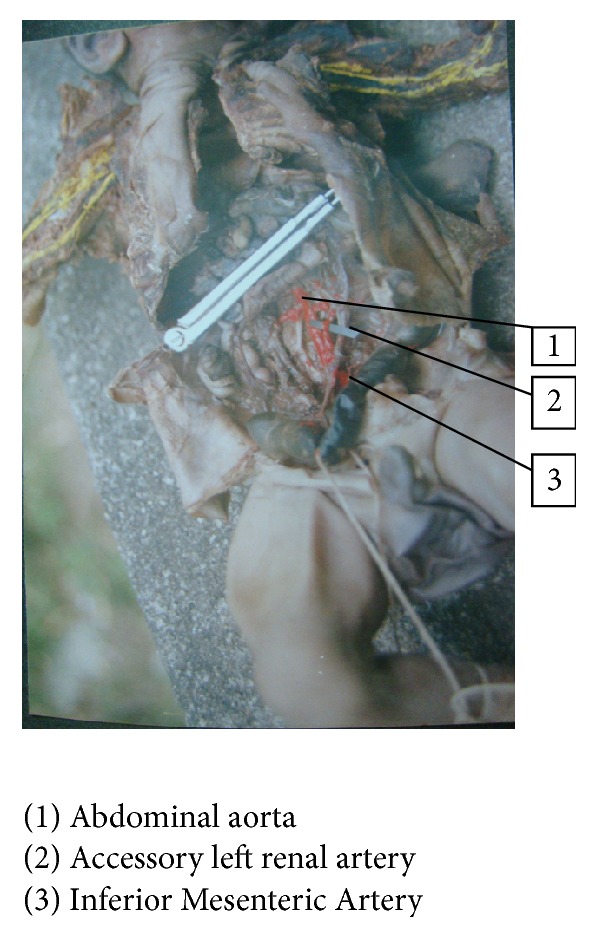
Accessory renal artery to left kidney from Inferior Mesenteric Artery.

**Figure 10:**
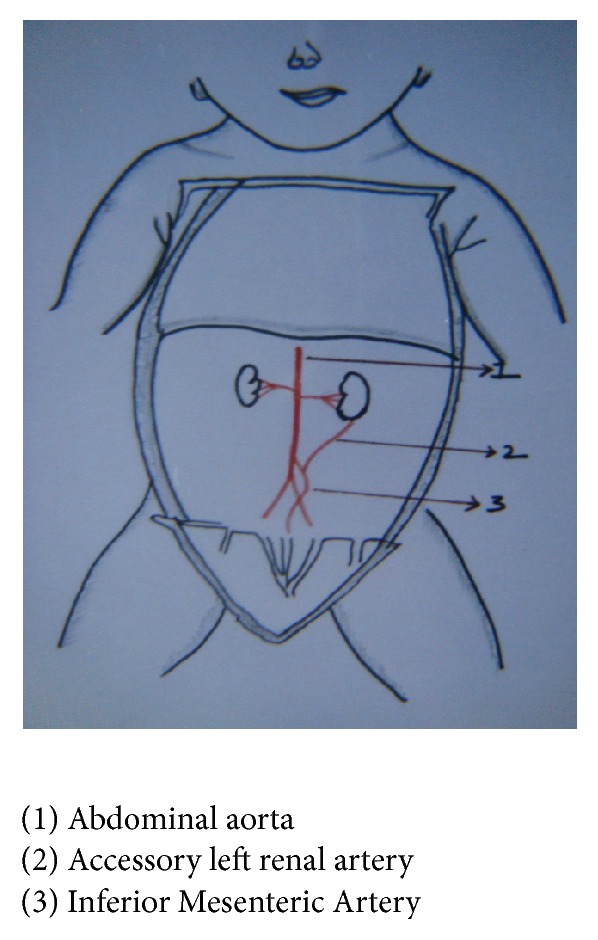
Accessory renal artery to left kidney from Inferior Mesenteric Artery, sketch.

In one fetus left renal artery was arising from Inferior Mesenteric Artery, 1.5%, fetus number 18: BPD—9.0 cms, age—35 weeks, and weight—1850 gms (Figures 7 and 8), and in another fetus, fetus number 51: BPD—8 cms, age—28 weeks, and weight—1850 gms (Figures 9 and 10), one accessory renal artery was arising from Inferior Mesenteric Artery and entering lower pole of left kidney.

*Results* are summarized in tabulated form in Tables 1 and 2.

## 4. Discussion

Embryologically ventral splanchnic arteries are paired and form capillary plexuses on the yolk sac. Inferior Mesenteric Artery is initially formed at twelfth thoracic vertebra but eventually migrated to the level of third lumbar vertebra [1]. Gross variations of the Inferior Mesenteric Artery have been reported infrequently earlier, though it is one of the essential splanchnic arteries. George [3] expressed that the artery rarely varies in its position, but he did not mention the number. In our study, only one fetus from group II (second trimester) had a high origin of Inferior Mesenteric Artery, that is, at first lumbar vertebra. Cauldwell and Anson [4] gave detailed embryological explanation regarding all the variations observed by him. This shift can be explained to be due to the growth and descent of organs supplied by the Inferior Mesenteric Artery [5].

The branches of Inferior Mesenteric Artery are the left colic artery, sigmoidal arteries (1 to 5), and superior rectal artery. Steward and Rankin [6] reported one to nine sigmoidal branches of Inferior Mesenteric Artery. Sunderland [7] found one to seven sigmoid branches arising from Inferior Mesenteric Artery among 25 adult specimens. Greenberg [8] in an investigation of 74 cases observed one to four sigmoid arteries.

In the present study, left colic artery was the first branch given off from the Inferior Mesenteric Artery, sigmoidal arteries were 1–5 in number, and superior rectal artery continued as the Inferior Mesenteric Artery into the pelvic cavity; except in one fetus, the left colic artery arose from the abdominal aorta, and in three fetuses Inferior Mesenteric Artery was giving a branch to left kidney among the 34 group I fetuses. Out of 66 fetuses in group II, 64 had normal branching. In one fetus left renal artery was arising from Inferior Mesenteric Artery, 1.5%, and in another fetus one accessory renal artery was arising from Inferior Mesenteric Artery and entering lower pole of left kidney. The origin of renal artery can arise from aorta or other vessels [9]. This can be explained by the embryological importance of ascent of kidney from the sacral region, with Inferior Mesenteric Artery giving main branch or accessory branches to the kidney.

The length and diameter of Inferior Mesenteric Artery were greater in group II than in group I fetuses. The arterial arrangements have been extensively studied by angiography and explanatory hypothesis has been advanced [10].

Recently Gangam and Lakmala in 2016 have reported that the most common branching pattern of Inferior Mesenteric Artery was into LCA in 74 (98.66%) cases and common sigmoid trunk which gave off 3-4 sigmoid arteries [11].

## 5. Conclusion

The course and branching pattern of Inferior Mesenteric Artery depend on the migration of organs.

## Figures and Tables

**Table 1:** Parameters of Inferior Mesenteric Artery.

Age group of fetuses	Average length in mms	Diameter at origin in mms	Site of origin
Second trimester	18–30 mm except in one fetus, 48 mm	0.5–1 mm	Third lumbar vertebra except in one specimen at first lumbar vertebra

Third trimester	30–34 mm	1-2 mm	Third lumbar vertebra

**Table 2:** Variations in the branches of Inferior Mesenteric Artery.

Age group of fetuses	Number of specimens studied	Number of specimens observed	Branches	%
Second trimester	34	(a) 30	(1) Left colic artery	88.2%
			(2) Sigmoidal arteries (1–5)	
			(3) Superior rectal artery	
		(b) 3	(1) Left colic artery	8.8%
			(2) Sigmoidal arteries (1–5)	
			(3) Superior rectal artery	
			(4) *Left renal artery*	
		(c) 1	(1) Sigmoidal arteries (1–5)	2.9%
			(2) Superior rectal artery	
			(3) *Left colic artery arising from abdominal aorta*	

Third trimester	66	(a) 64	(1) Left colic artery	96.9%
			(2) Sigmoidal arteries (1–5)	
			(3) Superior rectal artery	
		(b) 1	(1) Left colic artery	1.5%
			(2) Sigmoidal arteries (1–5)	
			(3) Superior rectal artery	
			(4) *Left renal artery*	
		(c) 1	(1) Left colic artery	1.5%
			(2) Sigmoidal arteries (1–5)	
			(3) Superior rectal artery	
			(4) *Accessory left renal artery*	
